# Supplementary material for: Understanding the Driving Forces That Trigger Mutations in SARS-CoV-2: Mutational Energetics and the Role of Arginine Blockers in COVID-19 Therapy
Source: Viruses. 2022 May 11;14(5):1029. doi: 10.3390/v14051029 (PMC9143829; doi:10.3390/v14051029)
Supplement: Supplementary file 1 [file viruses-14-01029-s001.zip › Supplementary material.pdf]

Table S1: Distances between RBD Residue/Atom and ACE2 Residue/Atom

RBD Residue/Atom	ACE2 Residue/Atom	Dist [Å]
_6lzg_1.b/^Y449/hh	a_6lzg_1.a/^D38/od1	1.837
_6lzg_1.b/^A475/o	a_6lzg_1.a/^S19/hg	1.835
_6lzg_1.b/^N487/hd22	a_6lzg_1.a/^Q24/oe1	2.217
_6lzg_1.b/^G496/o	a_6lzg_1.a/^K353/hz1	2.474
_6lzg_1.b/^Q498/he22	a_6lzg_1.a/^Q42/oe1	1.647
_6lzg_1.b/^T500/hg1	a_6lzg_1.a/^Y41/oh	1.753
_6lzg_1.b/^N501/hd21	a_6lzg_1.a/^Y41/oh	2.36
_6lzg_1.b/^G502/hn	a_6lzg_1.a/^K353/o	1.812

RBD Residue/Atom	ACE2 Residue/Atom	Dist [Å]
a_6lzg_1.b/^Y449/hh	a_6lzg_1.a/^D38/od1	1.837
a_6lzg_1.b/^A475/o	a_6lzg_1.a/^S19/hg	1.835
a_6lzg_1.b/^N487/hd22	a_6lzg_1.a/^Q24/oe1	2.217
a_6lzg_1.b/^G496/o	a_6lzg_1.a/^K353/hz1	2.474
a_6lzg_1.b/^Q498/he22	a_6lzg_1.a/^Q42/oe1	1.647
a_6lzg_1.b/^T500/hg1	a_6lzg_1.a/^Y41/oh	1.728
a_6lzg_1.b/^G502/hn	a_6lzg_1.a/^K353/o	1.812

Table S2: SARS-CoV-2 variants and dominating mutations

WHO name	Notable Mutations
Alpha	N501Y, E484K, K417N, P681H, D614G
Beta	K417N, E484K
Gamma	K417N, E484K, D614G
Delta	T478K, L452R, P681R, P614G, E484Q, Δ156-157, R158G, T19R, D950N
Epsilon	L452R
Kappa	L452R, E484Q, P681R
Lambda	L452Q
Omicron	E484A, T478K, K417N, N501Y, D614G, P681H, N679K
